# Bioinformatics Meets User-Centred Design: A Perspective

**DOI:** 10.1371/journal.pcbi.1002554

**Published:** 2012-07-12

**Authors:** Katrina Pavelin, Jennifer A. Cham, Paula de Matos, Cath Brooksbank, Graham Cameron, Christoph Steinbeck

**Affiliations:** EMBL-EBI, Wellcome Trust Genome Campus, Hinxton, Cambridge, United Kingdom; University of California San Diego, United States of America

## Abstract

Designers have a saying that “the joy of an early release lasts but a short time. The bitterness of an unusable system lasts for years.” It is indeed disappointing to discover that your data resources are not being used to their full potential. Not only have you invested your time, effort, and research grant on the project, but you may face costly redesigns if you want to improve the system later. This scenario would be less likely if the product was designed to provide users with exactly what they need, so that it is fit for purpose before its launch. We work at EMBL-European Bioinformatics Institute (EMBL-EBI), and we consult extensively with life science researchers to find out what they need from biological data resources. We have found that although users believe that the bioinformatics community is providing accurate and valuable data, they often find the interfaces to these resources tricky to use and navigate. We believe that if you can find out what your users want even before you create the first mock-up of a system, the final product will provide a better user experience. This would encourage more people to use the resource and they would have greater access to the data, which could ultimately lead to more scientific discoveries. In this paper, we explore the need for a user-centred design (UCD) strategy when designing bioinformatics resources and illustrate this with examples from our work at EMBL-EBI. Our aim is to introduce the reader to how selected UCD techniques may be successfully applied to software design for bioinformatics.

## Introduction: Designing for the User

UCD is a design philosophy where the requirements of users are taken into account at all stages of the design process for a service or product. The goal of UCD is to produce an effective and usable tool that is crafted for use by specific types of people. For example, in bioinformatics this could be on the web, a desktop application, or scientific instrumentation.

So what does UCD entail? Everyone is familiar with good user interfaces, whether they are using smart phones, listening to portable music devices or surfing the web. UCD involves placing the user at the forefront of your mind as you design, test, and implement your product. In fact, before you even know how the resource is going to look, you conduct user research to find out your users' needs, how they will use the tool, and what knowledge they have of the subject domain.

Put simply, if you design a product in consultation with the user, you will probably find that more people will use and benefit from your software. In the long term, easy-to-use interfaces cost less to produce and are easier to maintain. For example, Cooper [1] presents a business case that outlines how the end product will suffer if you do not apply UCD. It is also important to include users in the design process because it is difficult to predict exactly how they will interact with the software. Indeed, even after the product is launched, you should continue to think about your user community and their interactions with your resource.

## Bioinformatics Gets User-Friendly

UCD has rapidly developed into a well-respected field over the past two decades, with lots of research to support best practice [2]–[5]. The ubiquity of user-friendly interfaces may give the impression that UCD is everywhere, but it is relatively scarce in complex domains such as bioinformatics [6]. Javahery et al. [7] highlight that online bioinformatics resources often have interfaces that “lack the sophistication” of the websites and software that people come across in their daily lives. We have worked in bioinformatics for a number of years, and from our experience software developers tend not to focus on the usability of interfaces. In our view, there are several reasons for this. For instance, it is difficult to produce user-friendly interfaces for bioinformatics resources because the data that you are presenting are complex. You also need to find people with the right skills to carry out the usability testing (see Box 1).

**Box 1. User Experience Design as a Profession**

Are you interested in transitioning into a role within User Experience (UX)? Here are our tips:

- You are more likely to be successful as a UX professional if you enjoy working closely with others, have an outgoing personality, have a natural empathy for people, and can be persuasive in giving feedback. It is also important to understand and appreciate the needs of your users.
- Other skills include the ability to plan and manage your time, to be able to explain complex ideas to people who do not specialise in bioinformatics, to use your initiative when designing usability tests, and to have strong research skills.
- The role involves working closely with people who may not always support your work, and so you need to be able to handle criticism and communicate feedback in a sensitive manner.
- We advise reading about UX and keeping up to date with new techniques and technologies. For UX conferences and networking events, you could join the Usability Professionals' Association (http://www.upassoc.org/).
- It is not essential to take a formal degree to transition into UX; there are lots of short training courses on UCD practices, as well as part-time human computer interaction (HCI) qualifications.
- We believe that it is advantageous to have a bioinformatics background to carry out UCD work for bioinformatics resources: it allows you to tailor your UX activities to this niche audience.

There is also a lack of incentive: it is the novelty of the tool that gets the paper published, not the UCD work associated with it. Moreover, once the paper has been published, there may be less motivation to improve the tool. Another obstacle is the initial cost of investing in UCD for academic organisations, which lack a traditional business model. Although the users are the “customers” because they use the bioinformatics “products”, they do not pay for these services and do not have a direct say in what resources are funded. It is also more difficult to measure the impact that UCD has on research than it is to measure its effect on unit sales in a commercial environment. In user interviews, we have generally found that users accept poor usability of the resources because they are free, but ultimately the tools do not always provide what they really want.

Despite these difficulties, we would encourage all software developers in bioinformatics to try a UCD approach. The end users of bioinformatics resources are often bench biologists, who can find it difficult to access the information within these tools. Indeed, Bolchini et al. [8] conducted usability testing of bioinformatics resources, which demonstrated that usability problems (such as inconsistent navigation) prevented users from completing their tasks. Moreover, Bolchini found that users often struggle to hit on the “right” terminology when searching biological data repositories and they find the long list of results difficult to interpret. If the tools are hard to use, then the benefits of the resources cannot be realised. Conversely, if the software is easy to use and access, you are likely to see an increase in the number of users and citations, raising the profile of the resource and thus the team behind it. Ultimately, if scientists have greater access to the data, this provides more opportunities for scientific discovery.

Another reason to invest in UCD is that it could save you time and money in the long run: it is cheaper to ensure that the software is suited to the user at the start of a project than to pay for costly redesigns, maintenance, and user support further down the line. For example, Pressman [9] showed that for every $1 that you spend on solving a problem during the design phase, it would cost $10 to solve the same problem during the development phase and over $100 if you wait until after the product is released. This shows that by investing in UCD we can leverage the investment made in building these data services.

## Case Studies from EMBL-EBI

From our experience, UCD can help you to develop high quality bioinformatics resources. Several recent projects at EMBL-EBI have adopted a UCD approach, two of which we describe here. One project set out to redesign EMBL-EBI's main search service, EB-eye [10], with an initial focus on gene and protein information. Jenny Cham led the UCD work (see Author Profiles) and conducted user research interviews to find out how people retrieve the information they need from the Internet. Unsurprisingly, this showed that scientists often use Wikipedia and search engines such as Google. Users said that they would like the EBI website to provide a “biologically aware” search summary, so that if you searched a particular gene or protein, the results would summarise all of the information we have across each of our core resources in a single place. In response to this, the developers created an interactive prototype, and Jenny took this to one-to-one usability testing sessions with over 70 researchers from 20 institutions across Europe. Participants were from academic and industrial sectors. After several rounds of collecting feedback and refining the service, the new search was launched in January 2011. The user response has been so positive that the EBI has decided to expand the service to cover more scientific domains and to make it publicly available for any organisation to re-use as a stand-alone on their own websites.

In a parallel project, Jenny teamed up with Paula de Matos to develop an enzyme information portal. The brief for the portal was to provide a single gateway to all of EMBL-EBI's enzyme-related resources, including our databases and key web services. Paula and Jenny organised user workshops and interviews to find out what the users wanted from the site. After this, they tested prototypes with the users before writing any code to make sure the navigation and functionalities were optimal. Although the design phase was lengthy—nine months from the initial user research to the full technical specification—the development process is set to take only six months, rather than the usual two years with one full-time developer. Another advantage was that the developer actively participated in the usability testing, which helped her to know from an early stage what technology and tools she would need to create the resource. The enzyme portal launched in February 2012.

## Finding Out What Your Users Want

**Figure 1:**
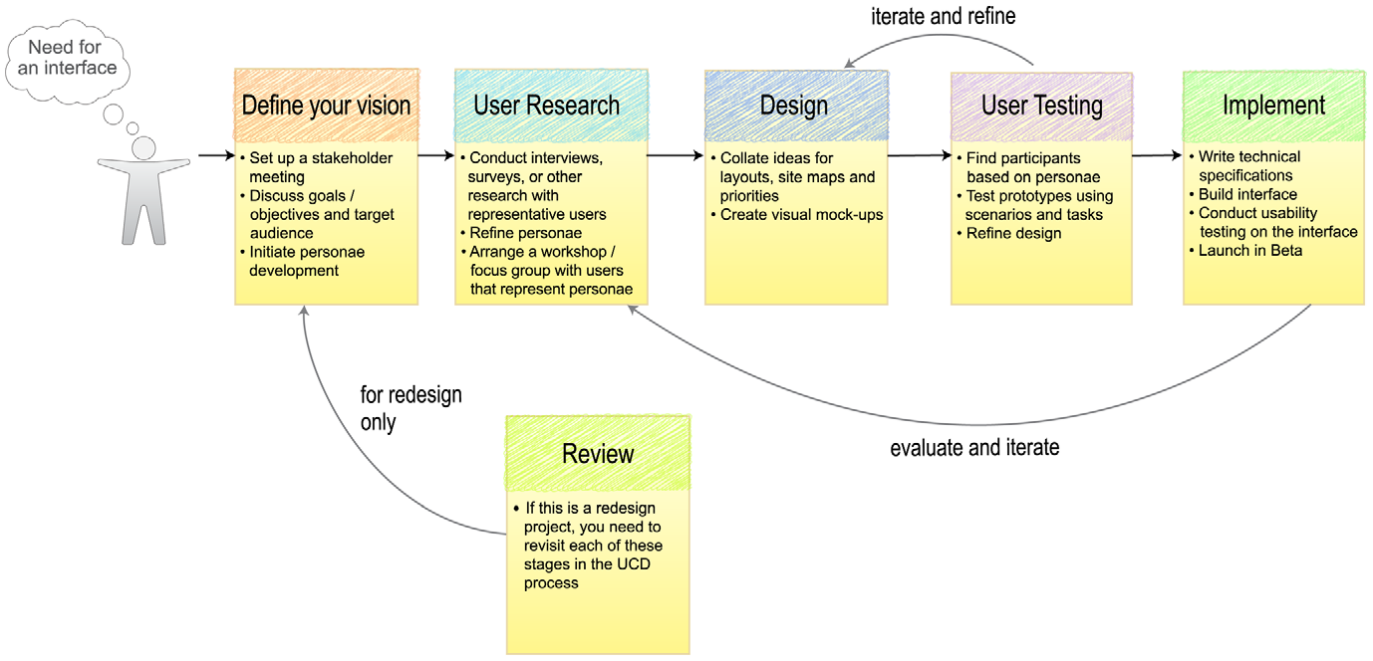
An overview of the user-centred design process. User-centred design focuses on the needs of the user, ensuring that the end product is fit for purpose. Once you have an idea for creating a new resource, or improving your existing resource, the key stages are: 1) Defining your goals and identifying your audience. 2) Characterising your users and their needs. 3) Designing mock-ups of your product. 4) Building prototypes of selected mock-ups and testing these with your users. 5) Writing the technical specifications and building the product. The UCD process is iterative; you continue to involve your users as you make improvements.

UCD may seem like a costly and time-consuming process, but basic usability testing is cheap and it can reveal how people perceive your resource. The ideal scenario would be to conduct user research before you design and implement your product. There are many UCD approaches that you could follow such as Contextual Design [11] or Participatory Design [12]; we have outlined an approach that has worked for us in Figure 1. If you have an existing resource, you could start by taking your laptop to the canteen and asking a colleague if they would take a look at your software. Set them a task—for example, finding a particular gene—and then watch what they do. You can also ask questions, such as “why did you click there?”, “is this what you were expecting to see?” If you do this with several people, and ideally record each session, you will gain insight into the usability of your prototype or product. If there are conflicting requirements you may need to do some deeper user research to understand this conflict. You may also need to prioritise user requirements, owing to resource constraints, technical considerations, and other organisational reasons. This should be done in consultation with users and stakeholders. Future iterations of the product can then incorporate any omitted user requirements.

But to get the most from UCD, you probably need some assistance from a User Experience (UX) professional. This could be someone working in-house or brought in as a consultant. The difficulty is that bioinformatics requires expert knowledge in multiple areas, so it can be challenging to find a UX specialist with a good understanding of both bioinformatics and UCD. We believe that it is preferable for a UX expert to be knowledgeable about the subject domain so they can design meaningful usability tests of biological data resources, but this is not an easy combination to find [6]. Another benefit of the UX person having domain knowledge is that it is easier for them to present their work persuasively, both to the users and to experts within the team. If the UX professional is not familiar with bioinformatics, they may benefit from bioinformatics training, or you could arrange for them to work closely with other bioinformaticians. Alternatively, software developers could train to be UX personnel, providing that they have a natural empathy towards users, as well as several other “soft skills” (Box 1). Essentially, it will take some effort to implement UCD techniques, but the benefits that UCD brings to the end product make it worthwhile.

## The User Is the Future

The UCD approach is growing in bioinformatics and more research teams are recruiting UX personnel. However, for bioinformaticians to adopt UCD more widely, there needs to be a culture shift across the field. Developers need the support of their group leaders to carry out UX work, which is not easy, given that UCD creates an initial delay in the design process. It is important to emphasise that UCD can actually speed up the development phase and ultimately results in products that are fit for purpose. For UCD to become more prevalent within bioinformatics, there also needs to be a funding incentive. One way to achieve this would be to demonstrate to funding organisations that UCD can help us to produce high quality bioinformatics resources. If UCD was a prerequisite for obtaining a grant, this could set a quality standard for biological data resources.

Another way to illustrate the value of user-focused databases to funding bodies would be to allocate a citation every time somebody uses a resource. This could act as a measure for the success of applying UCD techniques to bioinformatics resources, and it would give kudos to the developers. It would also help if researchers could publish their UX work, because this would raise the profile of UCD and demonstrate to funders that their resource is likely to attract users. Although the uptake of UCD in bioinformatics has been relatively slow, it is more widespread in other fields, so we can draw on lots of information about best practices. UCD may even change the direction of bioinformatics to areas that we did not realise were important. For example, we have found through user research that there is a lack of disease-centric resources in the public domain. If we allow the users to guide and inform the design process, they are more likely to use biological data resources, and this can only be a good thing for the progress of scientific understanding.

**Author Profiles**

**Katrina Pavelin** has a BA in Biology from the University of Oxford and an MSc in Science Communication from Imperial College London. She is currently the Scientific Outreach Officer at EMBL-EBI. Her role includes writing and editing for the web and print publications, both for users and the general public, as well as promoting the EBI at external events such as conferences.

**Paula de Matos** is a Group Coordinator and User Experience Analyst at EMBL-EBI. She was previously a software developer, but after realising the need to bridge the gap between the user community and the developer, she is undertaking a master's in Human Computer Interactions at University College London.

**Jennifer (Jenny) A. Cham** studied biochemistry at Imperial College London and has worked in pharmaceutical R&D. She has a master's and an engineering doctorate in bioinformatics from Cranfield University, United Kingdom, and now works as a User Experience Analyst at EMBL-EBI. Jenny is interested in usability practices in complex domains, including the application of user-centred design practices to support the development of user-friendly services for life scientists.

**Cath Brooksbank** is Head of Outreach and Training at EMBL-EBI. She has a PhD in biochemistry from the University of Cambridge and worked in the scientific publishing industry before joining EMBL-EBI. Her passion is enabling life scientists to make the most of biological data.

**Christoph Steinbeck** is Head of Cheminformatics and Metabolism at EMBL-EBI. His team develops databases and software infrastructures for small molecules and metabolism. While his group has been leading developments in open access and open source in cheminformatics in the past 10 years, he now realises that UCD might have made this a real success story.

**Graham Cameron** has recently retired from his role as Associate Director of EMBL-EBI. For the past 30 years he has worked for the European Molecular Biology Laboratory (EMBL), first in Heidelberg, and now in the UK. He was involved in the creation of EMBL-EBI and has been responsible for overseeing EMBL-EBI's operation since its conception.

## References

[1] Cooper A (2004). The inmates are running the asylum.

[2] Nielson J (1983). Usability engineering.

[3] Norman DA (2002). The psychology of everyday things. 2nd edition.

[4] Krug S (2005). Don't make me think: a common sense approach to web usability. 2nd edition.

[5] Buxton B (2007). Sketching user experiences: getting the design right.

[6] Chilana PK, Wobbrock JO, Ko JA (2010). Understanding usability practices in complex domains.. Proceedings of the 28th International Conference on Human Factors in Computing Systems (CHI '10).

[7] Javahery H, Seffah A, Radhakrishnan T (2004). Beyond power: making bioinformatics tools user-centered.. Comms ACM.

[8] Bolchini D, Finkelstein A, Perrone V, Nagl S (2009). Better bioinformatics through usability analysis.. Bioinformatics.

[9] Pressman RS (1992). Software engineering: a practitioner's approach.

[10] Valentin F, Squizzato S, Goujon M, McWilliam H, Paern J, Lopez R (2010). Fast and efficient searching of biological data resources – using EB-eye.. Brief Bioinform.

[11] Beyer H, Holtzblatt K (1998). Contextual design: defining customer-centered systems.

[12] Bodker S, Ehn P, Knudsen J, Kyng M, Madsen K (1988). Computer support for cooperative design.. In: Proceedings of the 1988 ACM conference on Computer-Supported Cooperative Work (CSCW '88).

